# Depressive symptoms are associated with reduced neutrophil function in hip fracture patients^☆^

**DOI:** 10.1016/j.bbi.2013.07.004

**Published:** 2013-10

**Authors:** Niharika Arora Duggal, Jane Upton, Anna C. Phillips, Peter Hampson, Janet M. Lord

**Affiliations:** a School of Immunity and Infection, University of Birmingham, Birmingham B15 2TT, UK; b MRC-Arthritis Research UK Centre for Musculoskeletal Ageing Research, University of Birmingham, Birmingham B15 2TT, UK; c School of Sport and Exercise Sciences, University of Birmingham, Birmingham B15 2TT, UK

**Keywords:** Ageing, Cortisol, Dehydroepiandrosterone, Depressive symptoms, Hip fracture, Neutrophil function

## Abstract

Hip fracture is a common trauma in older adults with a high incidence of depression, which relates to poorer prognosis including increased risk of infection. Ageing is accompanied by reduced immunity, termed immunesenescence, resulting in increased susceptibility to infection. We examined whether physical trauma (hip fracture) and psychological distress (depressive symptoms) had additive effects upon the aged immune system that might contribute to poor outcomes after injury. Neutrophil function was assessed in 101 hip fracture patients (81 female) 6 weeks and 6 months after injury and 43 healthy age-matched controls (28 female). Thirty eight fracture patients had depressive symptoms at 6 weeks. No difference in neutrophil phagocytosis of *Escherichia coli* was observed between controls and hip fracture patients, but superoxide production was significantly reduced in hip fracture patients with depressive symptoms compared with patients without symptoms (*p* = .001) or controls (*p* = .004) at 6 weeks. Superoxide production improved 6 months following fracture to the level seen in controls. We detected elevated serum cortisol, reduced dehydroepiandrosterone sulphate (DHEAS) and an increased cortisol:DHEAS ratio in fracture patients with depressive symptoms compared with patients without depressive symptoms or controls at 6 weeks and 6 months after injury. Serum IL6, TNFα and IL10 were higher among patients with depressive symptoms at 6 weeks. The cortisol:DHEAS ratio and IL6 levels related to depressive symptom scores but not to neutrophil function. In conclusion, depressive symptoms related to poorer neutrophil function after hip fracture, but this was not driven by changes in stress hormone or cytokine levels.

## Highlight

• Depressive symptoms are a major driver of reduced immunity after hip fracture.

## 1 Introduction

Hip fracture is a devastating condition and a major health issue in old age (Abrahamsen et al., 2009). In the UK alone 370,000 older adults fall each year and 76,000 of these falls result in hip fracture (Johnell et al., 1992). Even though hip fracture is treatable, it is a severe physical stressor for older individuals accompanied by increased mortality (Roberts and Goldacre, 2003), immobilisation and physical disability (Magaziner et al., 2000) resulting in loss of independence and impaired quality of life. The factors contributing to poor outcome after hip fracture remain poorly understood.

Healthy older individuals have been reported to experience greater levels of stress, anxiousness and depression than young adults (Luz et al., 2003). Stressful life events such as bereavement, or a disabling medical event are amongst the most potent factors that can trigger depressive symptoms (Cole and Dendukuri, 2003) and are both more likely to occur in old age. It is perhaps not surprising that a high rate of depression (9–47%) has been reported in UK and US based studies of older adults with hip fracture (Holmes and House, 2000). Importantly, depression in hip fracture patients has been associated with increased risk of infections and poor survival (Nightingale et al., 2001), impaired recovery and a retarded ability to regain pre-fracture levels of physical functioning (Mossey et al., 1990).

It is well documented that ageing is accompanied by immune dysregulation (Dorshkind et al., 2009; Panda et al., 2009), termed immunesenescence, which contributes to the increased risk of infection in old age (Gavazzi and Krause, 2002). Interestingly, there is accumulating evidence suggesting that the effects of stress and age are interactive, with chronic stress exacerbating the effects of ageing in older adults (Kiecolt-Glaser and Glaser, 1999). For example, a study examining the effect of the chronic stress of care giving on vaccine responses reported that even though there was a deficit in the vaccine response of young caregivers when compared with young controls, this defect was magnified in older adults who were caregivers (Kiecolt-Glaser et al., 1996). Additionally, our previous study reporting a negative association between exposure to stressful life events such as bereavement and marital dissatisfaction and antibody responses to vaccination, reported a significant positive effect of marital satisfaction on the response to vaccination in older adults (Phillips et al., 2006). Our own work has also shown that innate immunity is susceptible to the effects of stress, with neutrophil superoxide generation reduced in old hip fracture patients (Butcher et al., 2003) and bereaved older adults (Khanfer et al., 2011).

The hypothalamus–pituitary–adrenal (HPA) axis acts as a pivotal regulator of the stress responses by mobilising energy reserves and modulating immune responses (Tsigos and Chrousos, 2002). Glucocorticoids (GCs), namely cortisol in humans, are key effectors of the HPA axis and are potent immune suppressors. Dehydroepiandrosterone sulphate (DHEAS), a major steroid produced by the adrenal gland, has been reported to have anti-depressive, anti-glucocorticoid and immune-enhancing properties, including increased neutrophil superoxide generation (Hazeldine et al., 2010; Radford et al., 2010). Some previous studies have suggested that healthy ageing is accompanied by hyperactivation of the HPA axis, especially in situations of chronic stress resulting in prolonged exposure to cortisol (Aguilera, 2011). However, studies examining the effects of ageing on diurnal cortisol secretion have yielded conflicting results, with either a flattening of the diurnal pattern of secretion with increasing age (Deuschle et al., 1997; Luz et al., 2003; Van Cauter et al., 1996; Yen and Laughlin, 1998), no association (Edwards et al., 2001; Wolf et al., 2002), or decreased overall levels (Orentreich et al., 1992; Straub et al., 2000) with age. In contrast, the serum level of DHEAS reaches peak concentrations during the third decade of life, after which a steady decline occurs with age (1–2% per year); such that by the age of 80, DHEAS levels have reached 10–20% of their peak level (Vermeulen, 1995). Therefore, the current literature suggests that although serum cortisol may not increase markedly with ageing, cortisol levels are higher in relation to other hormones such as DHEAS and ageing is accompanied by an elevated cortisol:DHEAS ratio which may be a key factor contributing towards age associated immune dysregulation (Buford and Willoughby, 2008) and which might be heightened by chronic stress. In a previous study, our group reported a raised cortisol:DHEAS ratio in old hip fracture patients compared to comparable young trauma patients and this enhanced glucocorticoid response was also accompanied by reduced neutrophil superoxide generation and increased incidence of infection (Butcher et al., 2005). This study did not consider other factors that might influence immunity after hip fracture. Further, both ageing ((Franceschi et al., 2007; Krabbe et al., 2004)), and depression (Dowlati et al., 2010; Leonard, 2010; Zunszain et al., 2012) are accompanied by a switch towards a pro-inflammatory state, which can be seen in the levels of different pro- and anti-inflammatory cytokines. These cytokines have profound effects on immune cells and might also influence neutrophil function (Hellberg et al., 2011).

The present study sought to test the hypothesis that psychological distress, specifically depressive symptoms, would act additively with the physical stress of hip fracture to amplify the effect of ageing upon immunity (immunesenescence), with specific reference to neutrophil function. It also examined the role of the cortisol:DHEAS ratio and pro- and anti-inflammatory cytokines as potential mediators of any additive effects observed.

## 2 Methods

### 2.1 Participants

One hundred and one older hip fracture patients (two participants were excluded after consenting as it became apparent that they did not in fact meet the inclusion criteria) were recruited from five hospitals in Birmingham, UK between 2010 and 2012. Thirty-seven (26%) were male. Inclusion criteria were that participants had to be aged 60 years and over with a hip fracture sustained 4–6 weeks previously but with no chronic immune-related disorders e.g., cancer, diabetes, or taking any regular medications that might modify immunity, e.g., immunosuppressants, statins. Additionally patients must not have had any diagnosis of depression by a physician prior to age 50 years or be taking or have previously taken anti-depressant medication, in order to pick up patients with depressive symptoms emerging post-hip fracture rather than those with a prior history of and thus propensity to depression. Participants started on anti-depressant treatment, therapy, or any change that would mean they no longer met the inclusion criteria between week 6 and month 6 testing sessions were excluded. Forty-three healthy older adults, 17 male, (40%), were also recruited from the community as controls via invitation letters to the Birmingham 1000 Elders cohort of healthy older adults involved in current research at the University of Birmingham. These controls also had to meet the inclusion criteria above but not have a current hip fracture. The study was approved by South Birmingham Local Research Ethics Committee and all participants provided written informed consent (study ref: 09/H1203/80).

### 2.2 Study design and procedure

The study was a prospective case-control design with three groups of older adults: hip fracture patients with or without depressive symptoms and healthy older adults. Consent was gained whilst patients were still in hospital. All patients provided a blood sample and completed questionnaires and structured interviews 4–6 weeks and 6 months after hip fracture. Control participants attended the University, at the same time as we were sampling a hip fracture patient, for one-off blood sampling and completed a depression and anxiety symptoms scale and basic demographic information (see below) following the blood sample. Blood samples were taken between 09.00 and 11.00 to minimise any effect of diurnal variations in steroid levels. None of the participants had an acute infection at the time of blood sampling. Interviews were performed either in the hospital or in the patient’s home for hip fracture patients and at the university for control participants. Assays for neutrophil phagocytosis and superoxide production were performed on the same day as blood sampling. Serum was frozen for later hormone and cytokine analysis. Cytokines were only assessed at the six week time point.

### 2.3 Interview and questionnaires

Standard socio-demographic and health behaviour information were taken and all comorbidities and medications, prescription and over-the-counter, were recorded by the interviewer. The psychological status of the participant was assessed by means of standardised psychometric questionnaires. Depression was evaluated by a Geriatric Depression Scale (GDS) (Yesavage et al., 1982). Depression was defined as a GDS score greater than or equal to 6 (Sheikh and Yesavage, 1986). The Hospital Anxiety and Depression Scale (HADS) was also used to measure depression and anxiety (Zigmond and Snaith, 1983). The scale contains 14 items, scored from 0 (not present) to 3 (considerable), with seven assessing aspects of depression and seven assessing anxiety. Healthy control participants completed the HADS depression sub-scale in order to check that they did not have significant depressive symptoms. A cut-off of ⩾8 has previously been used to indicate possible depression (Bjelland et al., 2002).

### 2.4 Neutrophil functional assays

Neutrophil phagocytosis was measured in whole blood using a commercially available kit (Phagotest kit, Orpegen Pharma, Germany) and the assays were performed according to manufacturer’s instructions. Briefly, FITC-labelled opsonised *Escherichia coli* were added to whole blood and incubated at 37 °C for 10 min and the control tube was kept on ice for 10 min. Following incubation, quenching solution was added to each tube to stop the reaction and red blood cells were lysed. Following the lysis step the cell suspension was resuspended in DNA staining solution and fluorescence analysed immediately using a CyanTM ADP flow cytometer (Dako Ltd., Cambridge, UK). Data were analysed using Summit cV 4.3. The phagocytic index was used as a measurement of the phagocytic capabilities of neutrophils and was calculated as the percentage of cells that had ingested bacteria, multiplied by the mean fluorescence intensity (MFI), divided by 100. The coefficient of variation (CV) for the intra-assay variation was 5.1%.

Neutrophil superoxide burst was measured in whole blood using a commercially available kit (Phagoburst kit, Orpegen Pharma). The assays were performed according to manufacturer’s instructions. Briefly, whole blood was treated with 100 nM fMLP (Sigma Aldrich, Poole, UK), 20 nM PMA (Sigma Aldrich) or opsonised *E. coli* at 37 °C for 10 min. Following incubation, fluorogenic substrate dihydrorhodamine (DHR) 123 was added to the blood sample at 37 °C for ten minutes. Post incubation, cells were lysed after which DNA staining solution was added. Oxidation of substrate DHR123 was analysed by flow Cytometry using a CyanTM ADP flow cytometer. The oxidative burst production is indicated by the MFI of the neutrophil population. The coefficient of variation (CV) for the intra-assay variation was 3.2%.

### 2.5 Serum cortisol and DHEAS assays

Serum cortisol and DHEAS levels were measured by ELISA using a commercial kit (IBL international, Hamburg, Germany) according to manufacturer’s instructions. Intra assay coefficients of variation (CV%) were 6.7 for cortisol and 4.6 for DHEAS ELISAs.

### 2.6 Cytokine assays

A multiplex based assay for the cytokines IL1β, IL4, IL6, IL10, and TNFα (Bio-Rad Laboratories, Munich, Germany) was performed according to manufacturer’s instructions. Data acquisition and analysis was conducted using Bio-Plex Manager software version 6.0. Intra assay coefficients of variation (CV%) ranged from 7.15 to 13.89.

### 2.7 Statistical analysis

Univariate ANOVA with least significant difference post hoc tests were used to assess differences between the three groups (hip fracture with depressive symptoms, hip fracture without depressive symptoms, and healthy controls). Where demographic variables differed significantly between the three groups, analyses were rerun adjusting for these variables using ANCOVA. Pearson’s correlations were used to examine associations between depression score and neutrophil function and hormone levels. Where there were group differences in demographic or health behaviour variables, linear regression analyses were run with significant covariates entered at step 1. In order to test for potential mediation between depression and any effects on immune function by the cortisol:DHEAS ratio or cytokines, linear regressions were run predicting neutrophil function from hip fracture group entered at step 1 in the model, and the cortisol:DHEAS ratio or cytokine variable entered at step 2. Differences in degrees of freedom reflect occasional missing data. Data was missing where there was not sufficient serum for analyses, or the expense of multiple assays prohibited further analysis once the sample size needed by power analyses was complete (e.g., neutrophil phagocytosis, granulocyte numbers). In other cases, (e.g., cytokines) numbers also reflect lack of assay sensitivity for circulating non-stimulated cytokine levels in a relatively healthy sample. Patients in these reduced size analytic groups were a random sample of those tested, with the exception of neutrophil phagocytosis which was conducted on the first 116 participants as these are same day assays and sample sizes from power analyses are generally smaller for this assay.

## 3 Results

### 3.1 Recruitment and withdrawal

**Fig. 1:**
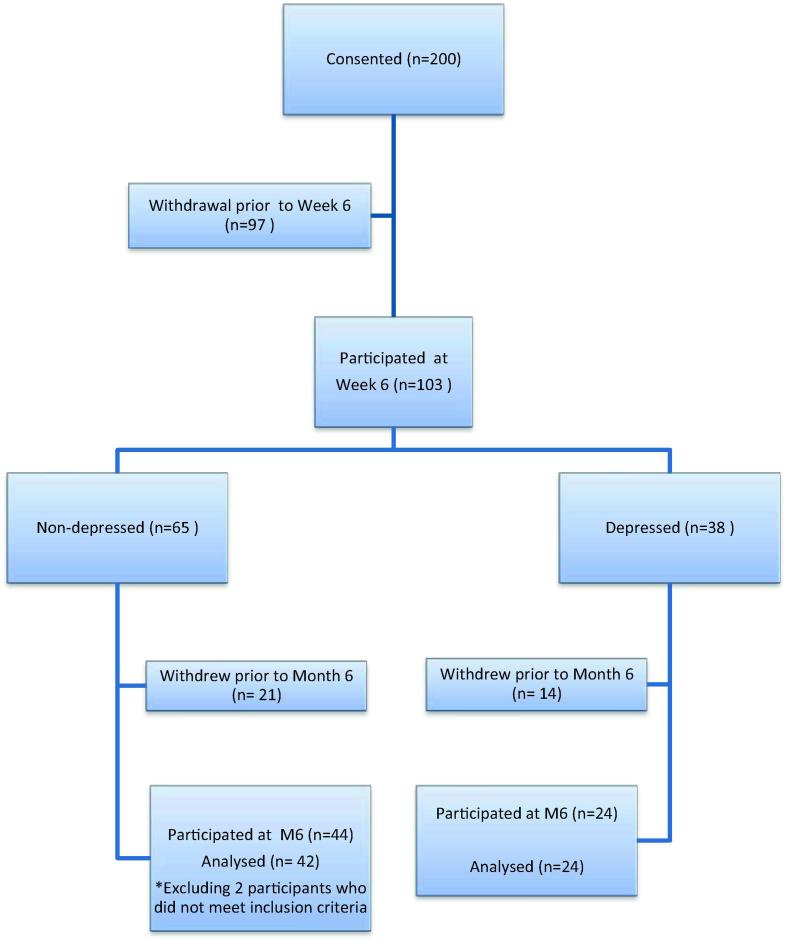
Recruitment and attrition in the study.

Recruitment and withdrawal data are shown in Fig. 1. Reasons for withdrawal between week 6 and month 6 included: death or being too unwell to be tested (*N* = 17), not being able to continue in the study for a variety of reasons including feeling they had too much to cope with, now receiving treatment for depressive symptoms or other medication/illnesses on the list of exclusion criteria, or being non-contactable (*N* = 18). There was little evidence of selection bias between the sample who withdrew or remained in the study at six months in terms of gender (*p* = .26), initial depression group status (*p* = .72), BMI (*p* = .34), or number of medications being taken (*p* = .09). However, those who withdrew were marginally more likely to be from the manual occupational group, (*p* = .05), were, on average, 3.9 years older (*p* = .02), and had 0.5 more comorbidities on average (*p* = .03).

### 3.2 Participant demographics

The demographic statistics and depression and anxiety scores for the study participants are shown in Table 1. Patients were classified into two groups on the basis of their GDS scores: hip fracture patients with a GDS score of 5 or less were classified as non-depressed (HF; hip fracture only), those with a score of 6 or greater were categorised as having significant depressive symptoms (HF + D; Hip fracture patients with depressive symptoms). In this study we observed that 38 (37%) of the hip fracture patients had depressive symptoms 4–6 weeks after their fracture. There were significant group differences between the controls and the hip fracture group for age and BMI, such that the control group were slightly younger, had a higher BMI, and more males. Consequently, all analyses were rerun with adjustment for age, BMI, and sex. As expected, the hip fracture group with depressive symptoms had significantly higher HADS depression scores than the hip fracture group without symptoms and the control group. However, there were no significant differences between the two hip fracture groups in terms of socio-economic status, smoking behaviour or alcohol consumption. At month 6, data were available for 66 hip fracture patients; 19 (29%) of whom were depressed.

### 3.3 Neutrophil count and function in hip fracture patients

**Fig. 2:**
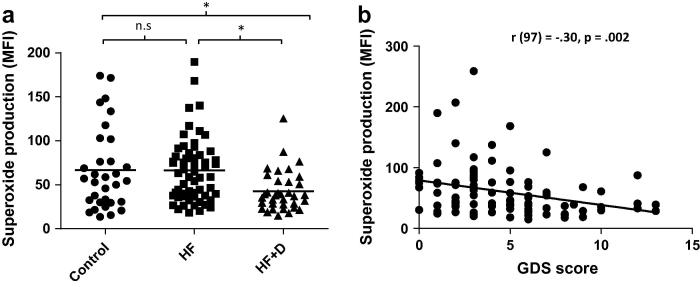
Neutrophil superoxide production on stimulation with *E. coli* in hip fracture patients with and without depressive symptoms versus healthy controls. (a) Neutrophil superoxide production (MFI) for hip fracture patients with (*n* = 35), or without depressive symptoms (*n* = 60) and healthy controls (*n* = 32). The solid bar represents the mean value and ^∗∗^indicates *p* < .005. (b) Correlation between GDS scores and neutrophil superoxide generation.

We compared granulocyte (mainly neutrophils) counts in peripheral blood of hip fracture patients with and without depressive symptoms with healthy older adults. There was a significant difference in the granulocyte count between the three groups in unadjusted analyses (see Table 2), such that the controls had significantly lower numbers of granulocytes than the hip fracture patients with (*p* = .04) and without depressive symptoms (*p* = .02), see Fig. 2a, though all were within the normal range. However, adjustment for covariates reduced the degrees of freedom due to missing data, thus this difference was no longer significant in analyses adjusted for age, BMI, and sex, see Table 3 for fully adjusted analyses data and effects of covariates.

Neutrophil phagocytosis in older adults did not differ between the three groups, although controls had slightly lower phagocytosis than hip fracture patients without depressive symptoms (*p* = .05).

Phagocytosis by neutrophils is followed by killing of ingested pathogens by production of reactive oxygen species (including superoxide anion) termed respiratory burst. Neutrophil superoxide production in response to PMA, in unadjusted and adjusted analyses, was significant between the three groups (see Tables 2 and 3). This was also the case for the response to opsonised *E. coli*. However, the significant impairment was most evident among the hip fracture patients who developed depressive symptoms: for PMA, *p* = .01, compared to controls, and *p* = .01, compared to hip fracture alone (data not shown), and for *E. coli*, *p* = .09 compared to controls and *p* = .003 compared to hip fracture alone. Fig. 2 shows the unadjusted analyses for *E. coli*.

### 3.4 Serum cortisol and DHEAS levels in hip fracture patients

**Fig. 3:**
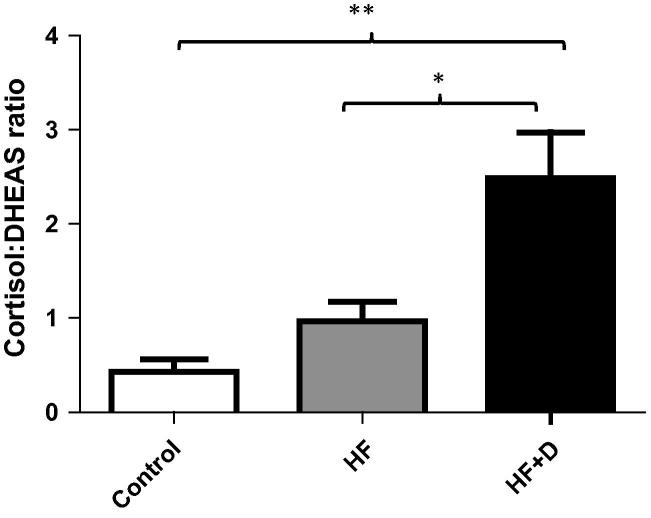
Stress hormone levels in hip fracture patients with and without depressive symptoms versus healthy controls. (a) cortisol: DHEAS ratio in hip fracture patients with (*n* = 35), or without depressive symptoms (*n* = 54) and healthy controls (*n* = 32). Data are mean ± SEM and for ^∗^*p* < .05, ^∗∗^*p* < .01 and ^∗∗∗^*p* < .001.

Analysis of HPA axis hormone levels revealed significant group differences at week six for serum cortisol in unadjusted and adjusted analyses (see Tables 2 and 3); similar results were found for DHEAS levels. This was driven by significantly higher cortisol in the hip fracture patients with depressive symptoms compared with patients with hip fracture alone, *p* < .001 and compared with controls, *p* = .04 (see Table 2). There was also lower DHEAS in the hip fracture patients with depressive symptoms compared with controls, *p* = .003, and those with hip fracture alone group, *p* = .05 (see Table 2). A significant group difference in the serum cortisol:DHEAS ratio was also observed, see Tables 2 and 3, such that only those hip fracture patients who developed depressive symptoms had a higher ratio when compared with non-depressed patients, *p* = .01 and they also had a higher ratio than those with hip fracture alone, *p* = .01. Fig. 3 shows the unadjusted values. However, there was no significant association between serum cortisol:DHEAS ratio and neutrophil superoxide production in the whole sample, *p* = .87, or in hip fracture patients alone, *p* = .71.

### 3.5 Serum cytokine levels in hip fracture patients

**Fig. 4:**
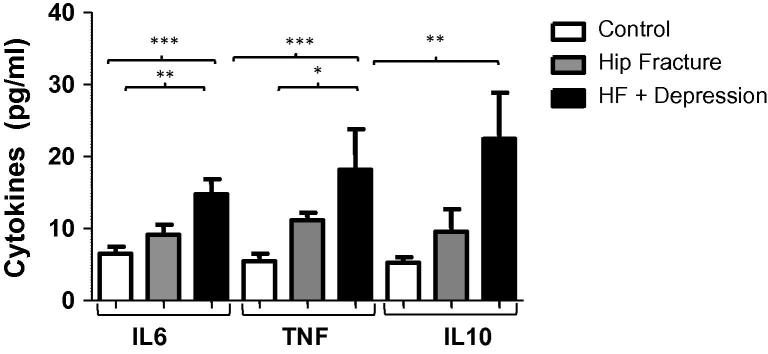
Serum cytokine levels in hip fracture patients with and without depressive symptoms versus healthy controls. (a) Mean serum IL6 levels for hip fracture patients with (*n* = 29), or without depressive symptoms (*n* = 37) and healthy controls (*n* = 28). (b) Mean serum TNFα levels for hip fracture patients with (*n* = 20), or without depressive symptoms (*n* = 21) and healthy controls (*n* = 23). (c) Mean serum IL10 levels for hip fracture patients with (*n* = 20), or without depressive symptoms (*n* = 21) and healthy controls (*n* = 23). Data are mean ± SEM and for ^∗^*p* < .05, ^∗∗^*p* < .01 and ^∗∗∗^*p* < .001.

On examining the serum cytokine levels in hip fracture patients, there were significant differences in serum IL6 levels between the three groups (Tables 2 and 3), such that a significant increase in serum IL6 levels was observed in hip fracture patients with depressive symptoms compared with non-depressed hip fracture patients (*p* = .001), and to healthy controls (*p* =.02) (Fig. 4a). However, IL6 did not correlate with neutrophil superoxide production, *p* = .24, thus showing no likely mediation of the association between depressive symptoms and reduced neutrophil function by IL6.

Next, on evaluating serum TNFα levels between the three groups (Tables 2 and 3), there was also a significant difference, such that higher serum TNFα levels were evident among the hip fracture patients with depressive symptoms compared with healthy controls (*p* < .001) and non-depressed hip fracture patients (*p* = .008) (Fig. 4b). Healthy controls also had lower TNFα than hip fracture patients without depressive symptoms (*p* = .04). There were no significant differences in serum IL1β levels between our three groups, *p* = .14 (see Table 2). Further, neutrophil function did not correlate with either TNFα (*p* = .47) or IL1β (*p* = .40), suggesting that these were not mediators of the association between depressive symptoms and reduced neutrophil function.

On examining the serum levels of anti-inflammatory cytokines, there were significant differences in serum IL10 levels between our three groups (see Tables 2 and 3), in adjusted analyses, such that significantly higher serum IL10 levels were shown among hip fracture patients with depressive symptoms compared to healthy controls (*p* = .006), but no difference in IL10 between hip fracture patients with depressive symptoms and those who were non-depressed (*p* = .08). Further, IL10 levels did not quite significantly correlate with neutrophil superoxide production (*p* = .06), thus there was no evidence of mediation of depressive symptom effects on neutrophil function by IL10. Finally, there were no significant differences in serum IL4 levels, *p* = .31, or in serum IL13 levels *p* = .40, between the three groups (see Table 2).

### 3.6 Sensitivity analyses

In order to check for associations with depression scores as a continuous variable rather than groups split at GDS = 6, regression analyses were performed in hip fracture patients. Similar to the ANCOVA results, in regression analysis, depressive symptom score itself did not predict granulocyte numbers, *β* = .01, *p* = .97, Δ*R*^2^ = .000. Further, as above, GDS score did not predict phagocytosis among hip fracture patients, *β* = .01, *p* = .91, Δ*R*^2^ = .000, but did predict lower neutrophil superoxide production in hip fracture patients to PMA, *β* = −.26, *p* = .05, Δ*R*^2^ = .066, and *E. coli, β* = −.30, *p* = .002, Δ*R*^2^ = .092, such that those with a greater symptom score had poorer superoxide production. Elevated serum cortisol:DHEAS ratio has been previously reported as a marker for depression (Young et al., 2002), and in the present study GDS scores, were significantly associated with the cortisol:DHEAS ratio, *β* = .25, *p* = .01, Δ*R*^2^ = .063, such that participants with greater depressive symptoms had a higher cortisol:DHEAS ratio, again driven by higher cortisol, *p* < .001, and lower DHEAS, *p* = .04, in those with more depressive symptoms. Interestingly, as shown in the ANCOVA model there was a significant association between GDS scores and IL6 levels in hip fracture patients, *β* = .29, *p* = .02, Δ*R*^2^ = .087, such that those with greater depressive symptoms had higher IL6. However, the regression model within the hip fracture patients were not significant for the association between depressive symptoms and TNFα, *β* = .19, *p* = .24, Δ*R*^2^ = .034. Again, as shown above, in hip fracture patients there were no associations with depressive symptoms and IL1β (*p* = .38), IL10 (*p* = .22), IL4 (*p* = .82) or IL13 (*p* = .20). Finally, regression analyses with continuous scores on the HADS depression subscale for the sample as a whole were also conducted, and the results were unchanged from those presented in the ANCOVA models above, with the exception that there was no longer a significant IL6 effect (details available on request from the authors).

### 3.7 Longer term effects of hip fracture and depressive symptoms

**Fig. 5:**
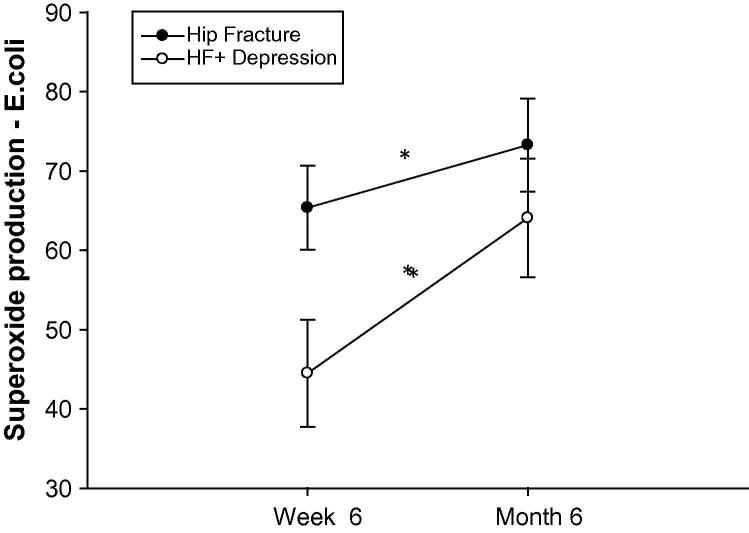
Neutrophil superoxide generation and cortisol:DHEAS ratio in hip fracture patients 6 weeks and six months after surgery. Mean neutrophil superoxide generation for hip fracture patients with (*n* = 26) or without depressive symptoms (*n* = 39) at 6 weeks and 6 months post injury. Data are mean ± SEM and ^∗^indicates *p* < .05.

The longer term effect of hip fracture and depressive symptoms on immune function was also evaluated. On examining neutrophil function in hip fracture patients six weeks and six months after injury, there was a significant main effect of time overall for hip fracture patients, F(1,56) = 6.83, *p* = .01, *η*^2^ = .109*,* such that superoxide production improved in both groups. There was also a main effect of group overall, F(1,56) = 4.12, *p* = .05, *η*^2^ = .068, such that the hip fracture patients with depressive symptoms had lower overall neutrophil function. However, the significant improvement in superoxide production over time in both groups meant that by six months, neutrophil function did not differ between the groups, *p* = .34, or relate to week 6 GDS score, *p* = .36, see Fig. 5.

On measuring serum hormone levels in hip fracture patients with depression six weeks and six months after injury there was no significant effect of time on the serum cortisol:DHEAS ratio, F(1,51) = 0.94, *p* = .34, *η*^2^ = .018, such that the ratio did not significantly alter overall. However, there remained a significant effect of group, F(1,51) = 7.54, *p* = .008, *η*^2^ = .129*,* such that the ratio was higher in the group with depressive symptoms overall and specifically at six months (*p* = .02) even though 11 of the previously depressed patients were now classified as non-depressed by six months. Correlation analyses with week 6 GDS score confirmed this, *r*(52) = .40, *p* = .003.

## 4 Discussion

In the present study, one third of patients had significant depressive symptoms post-hip fracture. These symptoms were associated with poorer neutrophil superoxide production and a higher cortisol:DHEAS ratio, as well as greater levels of the cytokines IL6 and TNFα and lower IL10, compared to healthy controls and hip fracture patients without depressive symptoms. This shows the potential impact of depressive symptoms which were independently related to immune function, rather than acting as an additive effect on top of hip fracture alone. However, the cortisol:DHEAS ratio and cytokine levels were not associated with neutrophil function, thus suggesting that depressive symptoms were independently associated with reduced superoxide production and that this is mediated through mechanisms other than those measured here.

The prevalence of depressive symptoms in the present study is in line with previous reports (Nightingale et al., 2001). Although mood disorders, especially depression, have been well studied in older patients with fractured neck of femur (Holmes and House, 2000; Lenze et al., 2007), this study has demonstrated for the first time that elevated depressive symptoms in these patients are associated with immune suppression. Crucially, neutrophil superoxide production was only impaired in those hip fracture patients that developed depressive symptoms; those with hip fracture alone showed neutrophil superoxide production equivalent to that of the healthy age-matched controls. This suppressed neutrophil superoxide production observed in hip fracture patients with depressive symptoms did not persist six months after injury and improved during that period.

We have previously reported that neutrophil superoxide production, specifically superoxide generation, was suppressed in older (>65 years) hip fracture patients, but not in younger trauma patients (Butcher et al., 2005). Moreover, we also reported that hip fracture was associated with a raised cortisol:DHEAS ratio that might mediate the effects of physical stress on neutrophil superoxide generation. However, in that study we did not determine the factors that were influencing the raised cortisol:DHEAS ratio and suppressed neutrophil function, thus we did not, for example, determine whether patients had depressive symptoms or not. The data described here reveal the important finding that depressive symptoms post-fracture in the absence of a prior history of depression appears to be strongly associated with reduced neutrophil function after injury. In addition we found that the altered levels of cortisol, DHEAS and their ratio were also only observed in the patients who developed depression and did not recover by six months post-fracture. Interestingly, we found an association between the cortisol:DHEAS ratio and depression score, but no association with reduced neutrophil superoxide generation, suggesting that factors other than altered HPA axis functioning are mediating the association between depression and neutrophil superoxide production.

Previous studies have reported an association between psychological wellbeing and HPA axis activity and increased cortisol levels is one of the most consistent findings in depressed patients (Burke et al., 2005; Gold et al., 1988), due to altered functioning of the glucocorticoid receptor (Zunszain et al., 2011). A decline in DHEAS levels in depressed patients has been reported to negatively correlate with a rating of depressed mood (Maninger et al., 2009). In the present study we observed an elevated serum cortisol:DHEAS ratio in hip fracture patients with depressive symptoms, due to increased serum cortisol levels and lower DHEAS levels, which is consistent with these previous studies.

It is well documented that ageing is accompanied by alterations in neutrophil bactericidal properties, including their ability to destroy ingested pathogens by superoxide generation (Panda et al., 2009; Wenisch et al., 2000). Emotional states such as depression are also known suppressors of immune function, for instance a study examining the effect of depression among children on neutrophil functioning has reported suppressed neutrophil superoxide generation with depression (Bartlett et al., 1997). In this study we have examined for the first time the additive effect of physical stress or trauma and psychological distress on immune dysregulation in older adults. Our findings are consistent with studies that have associated emotional stressors, such as bereavement in older adults with impaired neutrophil superoxide generation and failed to report an effect of such psychological distress on neutrophil phagocytic ability (Khanfer et al., 2011). Neutrophils play a prime role in defence against bacterial infections, thus this reduction in neutrophil superoxide production observed in hip fracture patients with depressive symptoms might increase susceptibility of infections in these patients, which may be a contributing factor towards ill-health and increased risk of mortality reported in these patients. However, the size of our study was not sufficient to verify this hypothesis even though 34.5% of the sample went onto develop infections.

One of the possible mechanisms usually thought to mediate reduced neutrophil superoxide production in depressed hip fracture patients could be altered activity of adrenal hormones, specifically DHEAS and cortisol. Cortisol is well documented as a suppressor of neutrophil superoxide generation (Bekesi et al., 2000; Butcher et al., 2005) and DHEAS enhances superoxide generation in *in vitro* assays (Radford et al., 2010). However our data suggest that although altered HPA axis activity was related to depression, it did not mediate the decrease in neutrophil function seen in depressed patients and another mechanism is providing the link between depression and reduced neutrophil superoxide generation. Assembly and activation of the NADPH oxidase complex involving association of cytosolic components p47phox, p67phox, and p40phox with membrane bound gp91phox and p22phox, catalyses electron transfer from NADPH to oxygen resulting in superoxide generation (Babior et al., 2002). A study examining the effect of academic psychological stress on neutrophil functioning reported that decreased superoxide production was accompanied by diminished expression of subunit p47phox of NADPH complex (Ignacchiti et al., 2011). Although not determined here, this may provide a mechanism for reduced superoxide generation in the depressed hip fracture patients.

Experimental and clinical evidence has suggested that depression and depressive symptoms are accompanied by elevated levels of pro-inflammatory cytokines, including IL6, TNFα and IL1β (Dowlati et al., 2010; Leonard, 2010; Zunszain et al., 2012). In this study, we have reported elevated levels of TNFα and IL6 in hip fracture patients with depressive symptoms compared with patients without depressive symptoms and healthy controls. This is consistent with findings of a previous study examining cytokine levels in hip fracture patients, according to which older hip fracture patients with high levels of pro-inflammatory cytokines such as IL6 had higher GDS scores (Matheny et al., 2011). Epidemiological studies have also reported an association between inflammatory marker IL6 and depressive symptoms in older people (Dentino et al., 1999; Penninx et al., 2003). Further, studies in animal models have shown that administration of pro-inflammatory cytokines are known to induce ‘sickness behaviour’ resulting in behavioural changes including sleep disturbance, loss of appetite, memory impairment and loss of interest in social activities that overlap with many of the symptoms of depression in humans (Dantzer et al., 2008; Kaster et al., 2012). A few studies conducted in older adults have suggested that inflammation might play a potential causal role in the development of depressive symptoms (Milaneschi et al., 2009; van den Biggelaar et al., 2007), suggesting that elevated levels of pro-inflammatory cytokines in certain hip fracture patients might increase the risk of development of depressive symptoms in this sub-group. As discussed previously, depression is associated with HPA axis hyperactivity, resulting in elevated cortisol levels. Interestingly, there is evidence to suggest that pro-inflammatory cytokines (IL6, TNFα, IL1β) are responsible for direct activation of the HPA axis (Dunn, 2000; Turnbull and Rivier, 1999). Thus, inflammation might be a driving factor for the development of depression. Indeed, in the present study, depressive symptoms score was significantly correlated with IL6 levels. However, TNFα, and IL1β did not correlate with depressive symptoms in the present study, nor did the hip fracture patient groups differ on these cytokines, other than for TNFα. Further, as these pro-inflammatory cytokines did not correlate with neutrophil superoxide production among hip fracture patients, while they may have played a role in the development of depressive symptoms, they did not mediate the association between depressive symptoms and neutrophil function or drive the differences in neutrophil function observed; depression was independently associated with neutrophil superoxide production.

Anti-inflammatory cytokines, such as IL10 are responsible for reducing production of pro-inflammatory cytokines and dampening immune responses (Moore et al., 2001). In this study, in addition to an elevation in levels of pro-inflammatory cytokines, including IL6 and TNFα, we have also reported an elevated serum level of anti-inflammatory cytokine IL10 in hip fracture patients with depressive symptoms compared to healthy controls but not non-depressed hip fracture patients. A similar elevation of IL10 has been previously reported in depressed individuals (Kubera et al., 2000; Simon et al., 2008), which might be a counter regulatory mechanism to try to reduce inflammatory responses and restore homeostasis. However, again as IL10 was not significantly associated with depressive symptoms score or neutrophil function in the present study, it is unlikely that it mediated the association between depression and neutrophil function or drove the effects on neutrophil function.

Hip fracture is associated with an increase in mortality that can persist for years after the fracture. Recovery after hip fracture is a long process and only one-third of patients have been reported to return to their pre-fracture functional status one year after the fracture (Magaziner et al., 2000). In an attempt to examine the long term effect of depression on immunity we examined neutrophil function in hip fracture patients with post-fracture depressive symptoms at six months after surgery. We observed that a higher cortisol:DHEAS ratio persisted at this time point but the neutrophil superoxide generation in hip fracture patients with depression had improved after 6 months. This further supports the proposal that the raised cortisol:DHEAS ratio did not mediate reduced neutrophil function in those with depressive symptoms after hip fracture.

The present study has several limitations. First, it was not possible to recruit a healthy population with depression symptoms to compare our results with, as any individuals that we might be able to identify from general practioner records would also be undergoing anti-depressant treatment, which was one of our exclusion criteria. Second, we were only able to assess cortisol and DHEAS at one time of day. Ideally, a diurnal assessment would be undertaken to give a more accurate fuller picture of changes in hormone secretion. However, in this frail patient group, it was not practical or ethical to increase the number of blood samples taken.

Despite reports regarding the high prevalence of depression and depressive symptoms in hip fracture patients it still remains undetected in the majority of patients and providing psychosocial care or pharmacological treatment for depression to these patients is rare. Recently, a nurse led intervention programme for older hip fracture patients with depression involving regular meetings with a psychiatric nurse and antidepressant medication proved to be a cost-effective method for reducing depressive symptoms (Romeo et al., 2011). Antidepressants, in addition to attenuation of depressive symptoms, have also been reported to reduce neurological disturbances observed in depressed patients, such as HPA axis hyperactivity by enhancing GR-mediated negative feedback by glucocorticoids by increasing GR expression and function. In addition to clinical interventions, proper counselling, psychological support, broadening social involvement (Cohen et al., 1997) and maintaining healthful practises (proper diet, exercise) may appease the deleterious effects of chronic exposure to stress and preserve immune function.

In conclusion, the present study reports for the first time that development of depressive symptoms in older hip fracture patients can result in suppression of neutrophil bactericidal properties, changes in circulating levels of certain and long term altered adrenocortical hormone balance. These findings suggest that development of depressive symptoms after a hip fracture in older adults is the main driver of immune suppression, as we failed to find an immune decline as a result of hip fracture alone. Our findings support the need for preventing and treating depression and depressive symptoms to improve outcomes in older hip fracture patients.

## Figures and Tables

**Table 1:** Participant demographics and questionnaire scores.

Variable	Mean (SD)/*N* (%)			*p*
	Controls	Hip fracture patients (HF)	Hip fracture patients with depressive symptoms (HF + D)	
*N* at week 6	43	65	38	
Age	74.9 (5.64)	83.8 (7.48)	84.0 (8.62)	<.001
BMI	27.6 (5.02)	23.5 (3.81)	22.7 (4.03)	<.001
Sex: male	17 (40)	13 (21)	7 (18)	.05
Occupational group: manual	–	28 (43)	21 (58)	.21
Smokers	2 (8)	7 (11)	6 (19)	.60
Alcohol consumption: one or more units per week	13 (50)	24 (37)	13 (34)	.43
Number of co-morbidities	–	2.2 (1.41)	2.0 (1.28)	.40
Number of medications	–	4.6 (2.68)	5.3 (2.96)	.23
GDS	–	2.6 (1.53)	8.2 (2.47)	<.001
HADS depression	2.4 (1.71)	3.4 (2.31)	9.1 (4.71)	<.001
HADS anxiety	–	4.0 (3.71)	8.0 (4.46)	<.001
N at month 6	–	47 (71)	19 (29)	.72
GDS at month 6	–	2.8 (1.90)	8.7 (2.67)	<.001

**Table 2:** Participant immune data by group (unadjusted analyses).

Variable	Mean (SD)			Statistics
	Controls	HF	HF + D	
Granulocyte count (10^9^/L)	3.67 (1.29)	4.84 (1.77)	4.86 (1.68)	F(2,66) = 4.76,*p* = .01, *η*^2^ = .12
Phagocytosis (MFI)	207.27 (46.07)	251.64 (98.86)	241.66 (123.69)	F*(*2,112) = 2.29,*p* = .11, *η*^2^ = .039
Superoxide production to PMA (MFI)	101.11(62.95)	91.03 (64.60)	50.46 (29.75)	F(2,87) = 5.53, *p* = .005, *η*^2^ = .113
Superoxide production to *E. coli* (MFI)	68.50 (39.16)	68.87 (43.36)	42.84(22.99)	F(2,134) = 6.52, *p* = .002, *η*^2^ = .089
Serum cortisol (mg/mL)	.130 (.05)	.121 (.04)	.169 (.05)	F(2,127) = 10.32, *p* < .001, *η*^2^ = .140
Serum DHEAS (mg/mL)	.814 (.367)	.429 (.792)	.184 (.157)	F(2,127) = 10.47, *p* < .001, *η*^2^ = .14
Cortisol:DHEAS ratio	.288 (.469)	.994 (1.67)	2.22(2.81)	F(2,127) = 9.36, *p* < .001, *η*^2^ = .12
IL6	6.25 (6.40)	7.31 (5.15)	13.79 (9.75)	F(2,101) = 10.25, *p* < .001, *η*^2^ = .169
TNFα	4.28 (4.58)	9.96 (6.06)	19.25 (19.52)	F(2, 62) = 9.52, *p* <.001, *η*^2^ = .235
IL1β	18.41 (13.77)	12.84 (7.92)	26.29 (29.62)	F(2,58) = 2.05, *p* = .14, *η*^2^ = .066
IL10	5.27(5.34)	10.37 (10.15)	22.41 (28.91)	F(2,66) = 7.25, *p* = .001, *η*^2^ = .180
IL4	2.37 (2.21)	1.45 (3.60)	3.37 (9.64)	F(2,89) = 0.82, *p* = .44, *η*^2^ = .018
IL13	16.48 (2.67)	7.47 (4.02)	15.84 (3.77)	F(2,63) = 1.86, *p* = .16, *η*^2^ = .056

**Table 3:** Adjusted analyses for main significant outcomes.

Dependent Variable	Variables in Model	(df)	F	*p*	*η*^2^
Granulocyte count					
	Age		0.04	.84	.001
	BMI		1.18	.28	.021
	Sex		0.26	.62	.005
	Depression group	2,55	1.62	.21	.056
Phagocytosis	Age		3.72	.06	.036
	BMI		1.97	.16	.019
	Sex		0.12	.73	.001
	Depression group	2,100	2.11	.13	.041
Superoxide production to PMA	Age		3.04	.09	.041
	BMI		0.03	.86	.000
	Sex		1.97	.17	.027
	Depression group	2,71	4.75	.01	.118
Superoxide production to *E. coli*	Age		1.80	.18	.015
	BMI		3.84	.05	.031
	Sex		0.12	.73	.001
	Depression group	2,120	4.56	.01	.071
Serum cortisol (mg/mL)	Age		0.28	.60	.003
	BMI		0.73	.39	.007
	Sex		2.23	.14	.020
	Depression group	2,111	7.72	.001	.122
Serum DHEAS (mg/mL)	Age		3.86	.05	.034
	BMI		0.46	.50	.004
	Sex		4.65	.03	.040
	Depression group	2,111	4.80	.01	.080
Cortisol:DHEAS ratio	Age		0.11	.75	.001
	BMI		1.33	.25	.012
	Sex		0.75	.39	.007
	Depression group	2,110	5.00	.008	.083
IL6	Age		6.72	.01	.072
	BMI		0.05	.82	.001
	Sex		3.17	.08	.035
	Depression group	2,87	6.71	.002	.134
TNFα	Age		4.51	.04	.078
	BMI		0.30	.58	.006
	Sex		2.99	.09	.053
	Depression group	2,53	8.04	.001	.233
IL10	Age		3.34	.07	.059
	BMI		1.19	.28	.022
	Sex		0.32	.58	.006
	Depression group	2,53	4.43	.02	.143
